# Improvement of Quantification of Myocardial Synthetic ECV with Second-Generation Deep Learning Reconstruction

**DOI:** 10.3390/jcdd11100304

**Published:** 2024-10-02

**Authors:** Tsubasa Morioka, Shingo Kato, Ayano Onoma, Toshiharu Izumi, Tomokazu Sakano, Eiji Ishikawa, Shungo Sawamura, Naofumi Yasuda, Hiroaki Nagase, Daisuke Utsunomiya

**Affiliations:** 1 Department of Radiology, Yokohama City University Hospital, Yokohama 236-0004, Kanagawa, Japan; morioka@yokohama-cu.ac.jp (T.M.); onoma@yokohama-cu.ac.jp (A.O.); izumi3@yokohama-cu.ac.jp (T.I.); sakanotm@yokohama-cu.ac.jp (T.S.); cqa10233@yokohama-cu.ac.jp (E.I.); 2 Department of Diagnostic Radiology, Yokohama City University Graduate School of Medicine, Yokohama 236-0004, Kanagawa, Japan; sawa0808@yokohama-cu.ac.jp (S.S.); yasuda.nao.mz@yokohama-cu.ac.jp (N.Y.); nagase.hir.pc@yokohama-cu.ac.jp (H.N.); d_utsuno@yokohama-cu.ac.jp (D.U.)

**Keywords:** synthetic ECV, deep leaning reconstruction, computed tomography, myocardial fibrosis

## Abstract

Background: The utility of synthetic ECV, which does not require hematocrit values, has been reported; however, high-quality CT images are essential for accurate quantification. Second-generation Deep Learning Reconstruction (DLR) enables low-noise and high-resolution cardiac CT images. The aim of this study is to compare the differences among four reconstruction methods (hybrid iterative reconstruction (HIR), model-based iterative reconstruction (MBIR), DLR, and second-generation DLR) in the quantification of synthetic ECV. Methods: We retrospectively analyzed 80 patients who underwent cardiac CT scans, including late contrast-enhanced CT (derivation cohort: *n* = 40, age 71 ± 12 years, 24 males; validation cohort: *n* = 40, age 67 ± 11 years, 25 males). In the derivation cohort, a linear regression analysis was performed between the hematocrit values from blood tests and the CT values of the right atrial blood pool on non-contrast CT. In the validation cohort, synthetic hematocrit values were calculated using the linear regression equation and the right atrial CT values from non-contrast CT. The correlation and mean difference between synthetic ECV and laboratory ECV calculated from actual blood tests were assessed. Results: Synthetic ECV and laboratory ECV showed a high correlation across all four reconstruction methods (R ≥ 0.95, *p* < 0.001). The bias and limit of agreement (LOA) in the Bland–Altman plot were lowest with the second-generation DLR (hybrid IR: bias = −0.21, LOA: 3.16; MBIR: bias = −0.79, LOA: 2.81; DLR: bias = −1.87, LOA: 2.90; second-generation DLR: bias = −0.20, LOA: 2.35). Conclusions: Synthetic ECV using second-generation DLR demonstrated the lowest bias and LOA compared to laboratory ECV among the four reconstruction methods, suggesting that second-generation DLR enables more accurate quantification.

## 1. Introduction

Myocardial fibrosis is a histopathological change observed in various heart diseases, and accurate assessment is crucial for diagnosis [1]. Cardiac magnetic resonance imaging (MRI) is an established imaging modality that can non-invasively evaluate histological changes in the myocardium, and it measures indicators such as late gadolinium enhancement and extracellular volume fraction (ECV) [2,3,4], both of which are highly correlated with myocardial fibrosis. However, cardiac MRI has limitations, including long scan times, high costs, and various contraindications such as claustrophobia and the presence of metallic devices. Recently, the assessment of myocardial ECV using CT imaging has gained attention, and its reliability is considered comparable to MRI-ECV [5]. Cardiac CT exams are performed far more frequently than MRI, making CT-ECV more accessible in clinical practice. CT-ECV is particularly useful in detecting cardiac amyloidosis associated with aortic stenosis. Approximately 15% of patients undergoing preoperative evaluation for transcatheter aortic valve implantation have coexisting cardiac amyloidosis and aortic stenosis [6].

However, measuring CT-ECV requires the measurement of hematocrit values. Hematocrit measurement is invasive and subject to significant fluctuations depending on the timing of the test, which can be a barrier to the widespread use of CT-ECV. Recently, the utility of synthetic ECV, which does not require hematocrit measurement, has been reported [7,8]. Due to its convenience, synthetic ECV has the potential to significantly promote the clinical use of CT-ECV. While synthetic ECV has been reported to correlate well with conventional ECV, high-quality cardiac CT images with minimal noise are essential for accurate calculation.

Artificial intelligence-driven algorithms offer the advantage of simultaneously achieving noise reduction and preserving image details with high accuracy compared to conventional statistical iterative and model-based iterative reconstruction techniques. Precise IQ Engine (PIQE), a second-generation deep learning reconstruction (DLR) technology, uses high-resolution CT images as training data to achieve both high resolution and noise reduction [9,10]. Previously, we conducted an experiment using a coronary artery phantom to evaluate the image quality of PIQE and demonstrated that PIQE improved visual assessment indicators, such as vascular sharpness, granularity, and visibility, compared to conventional reconstruction methods [11]. To date, there have been no reports on the utility of synthetic CT-ECV using DLR technology.

In this study, we compared the utility of PIQE, a second-generation DLR technology, with conventional reconstruction methods in the calculation of synthetic ECV using cardiac CT.

## 2. Materials and Methods

This study is a retrospective analysis of cardiac CT data from a single institution. The inclusion criteria consisted of consecutive cases in which comprehensive cardiac CT, including late contrast-enhanced CT, was performed based on clinical indications. The exclusion criteria included cases with poor image quality on cardiac CT, cases where misregistration occurred between non-contrast CT and late contrast-enhanced CT images due to arrhythmias, and cases without blood test data within 20 days of the CT examination. The derivation cohort consisted of 40 patients, and a regression equation for calculating synthetic hematocrit was established. The validation cohort comprised a different set of 40 patients, distinct from the derivation cohort, and synthetic ECV was calculated using the synthetic hematocrit derived from the regression equation of the derivation cohort. This synthetic ECV was then compared with the laboratory ECV calculated from the hematocrit obtained through blood tests. This single-center retrospective study was approved by the ethics committee of Yokohama City University Hospital (approval number: F240800006). Informed consent was obtained from all participants using the opt-out approach.

### 2.1. Cardiac CT Imaging Protocol

For all data collection, the imaging protocol utilized a Volume Scan. The basic protocol included non-contrast CT for calcium scoring, coronary CTA, and late contrast-enhanced CT. A low-dose non-contrast scan was performed for subtraction in ECV measurement. Data were collected approximately one minute after contrast agent injection, followed by additional data collection six minutes after contrast administration. The cardiac CT (CCT) examinations were conducted using a 320-row CT scanner (Canon Aquilion ONE PRISM edition: Canon Medical Systems Corporation, Tochigi, Japan), covering the area from 15 mm above the left main coronary artery to 15 mm below the inferior margin of the heart. Imaging conditions were set at a tube voltage of 120 kVp, with tube current controlled by CT-AEC (Automatic Exposure Control), a slice thickness of 0.5 mm, and a rotation speed of 0.275 s. ECG synchronization was performed with data acquisition at 70–80% of the R-R interval for heart rates of 70 bpm or below and 40–50% for bpm of 71 or above.

The contrast agents used were Iopamidol 300 (Bayer, Leverkusen, Germany) and Iomeprol 350 (Bracco Japan, Tokyo, Japan), injected at a rate of 24.5 mgI/kg/sec, resulting in a total administration of approximately 500 mgI/kg. After data acquisition, the non-contrast and 6 min post-contrast data were reconstructed using HIR (FC43 Standard), MBIR (Cardiac Standard), DLR (Cardiac Standard), and second-generation DLR (Cardiac Standard), maintaining the same center of the field of view and the same phase.

### 2.2. Calculation of Synthetic Hematocrit

In the derivation cohort, synthetic hematocrit values were calculated from non-contrast imaging by analyzing the data from the non-contrast scan used for calcium scoring. The following four types of reconstructed images were used: HIR, MBIR, DLR, and second-generation DLR. The region of interest measurements were taken on a slice centered in the right atrium and aligned with the height of the intervertebral disk, with a diameter of 6 cm to reduce partial volume effects. This slice was selected to minimize the impact of beam-hardening effects caused by the spine. As in the previous study, a linear regression line was derived to calculate synthetic hematocrit [7].

### 2.3. Calculation of Synthetic ECV

**Figure 1:**
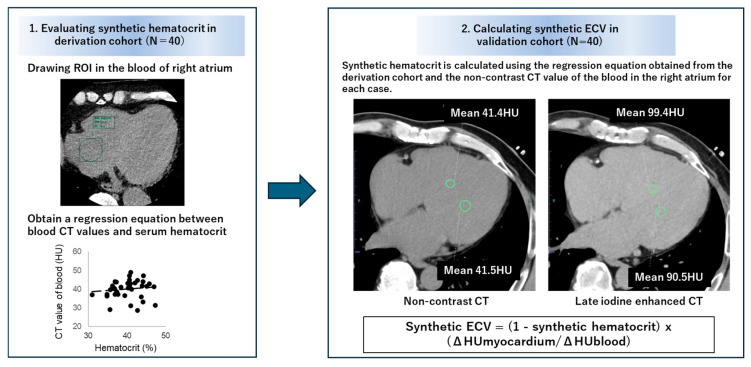
Methodology for calculating synthetic extracellular volume fraction.

In the validation cohort, using the non-contrast CT images for calcium scoring and late contrast-enhanced CT images, CT values of the right atrial cavity and the interventricular septum of the left ventricle were measured in the axial images, and the difference (Δ Hounsfield unit (HU) value) was calculated (Figure 1). Synthetic hematocrit was estimated using the previously mentioned linear regression equation, and synthetic ECV was calculated using the following formula.

Synthetic ECV = (1 − Synthetic hematocrit) × (ΔHU myocardium/ΔHU blood)(1)

The measurement of ECV using hematocrit values obtained from blood tests was performed using the following formula.

Laboratory ECV = (1 − Hematocrit) × (ΔHU myocardium/ΔHU blood)(2)

To evaluate inter-observer reproducibility, the measurement of second-generation synthetic ECV in 20 patients was conducted by another independent observer.

### 2.4. Statistical Analysis

Statistical analysis was performed using SPSS Statistics version 29 (IBM, Armonk, NY, USA) and MedCalc^®^ Statistical Software version 20.010 (MedCalc Software Ltd., Ostend, Belgium; https://www.medcalc.org (accessed on 1 October 2024)). In the derivation cohort, the correlation between the CT values of the right atrium on non-contrast CT and the hematocrit values obtained from blood tests was evaluated using Spearman’s correlation coefficient, and a regression equation was derived for calculating synthetic hematocrit. In the validation cohort, the correlation between synthetic ECV and laboratory ECV was analyzed using Spearman’s correlation coefficient, and the bias and limits of agreement (LOA) between the two were evaluated using the Bland–Altman method. To assess the interobserver reproducibility of measurement of synthetic ECV, the intraclass correlation coefficient (ICC) was evaluated. Statistical significance was defined as *p* < 0.05.

## 3. Result

### 3.1. Patients’ Characteristics

The characteristics of the patients in the derivation cohort (*n* = 40) and the validation cohort (*n* = 40) are presented in Table 1. In both cohorts, over half of the patients were male, with an average age of 71 ± 12 years for the derivation cohort and 67 ± 11 years for the validation cohort.

Atrial fibrillation was observed in two patients in the derivation cohort only. Renal function was generally well preserved, with an average estimated glomerular filtration rate (eGFR) above 60 mL/min/m^2^, and no patients had severe renal impairment (eGFR < 30 mL/min/m^2^).

### 3.2. Creation of the Regression Equations for Synthetic Hematocrit Calculation in the Derivation Cohort

**Figure 2:**
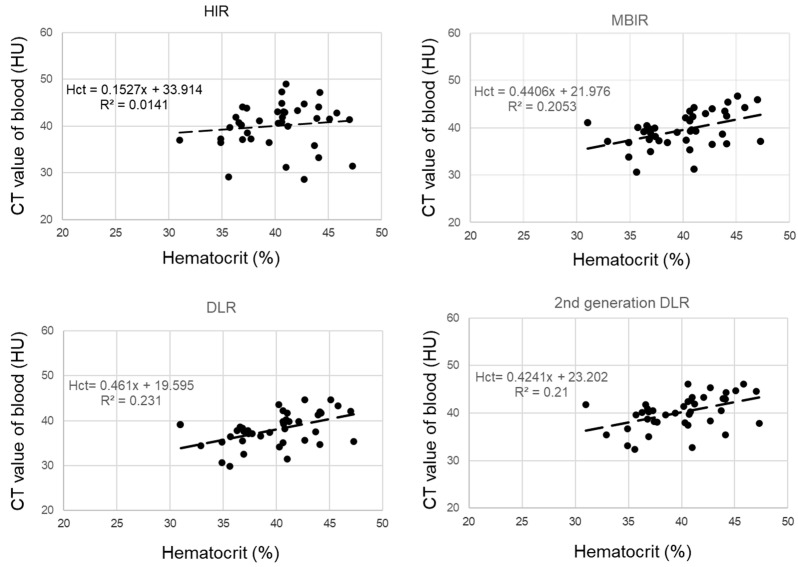
Correlation between blood CT value and serum hematocrit value in the derivation cohort.

The derivation cohort consisted of 40 cases, with 63% being male and an average age of 67 ± 11.1 years. Hematocrit measurements were conducted within 20 days (average 8 days) of the CT scan. The regression equations for calculating synthetic hematocrit across the four reconstruction methods were determined as follows (Figure 2):

HIR: synthetic hematocrit = (0.15 × laboratory hematocrit) + 33.9, (R^2^ = 0.01) (3)

DLR: synthetic hematocrit = (0.42 × hematocrit) + 23.2, (R^2^ = 0.23) (4)

Second-generation DLR: synthetic hematocrit = (0.46 × hematocrit) + 19.6, (R^2^ = 0.21) (5)

These regression lines were used to estimate synthetic hematocrit for each reconstruction method in the validation cohort.

### 3.3. Comparison of Synthetic ECV and Laboratory ECV across Four Reconstruction Methods in the Validation Cohort

**Figure 3:**
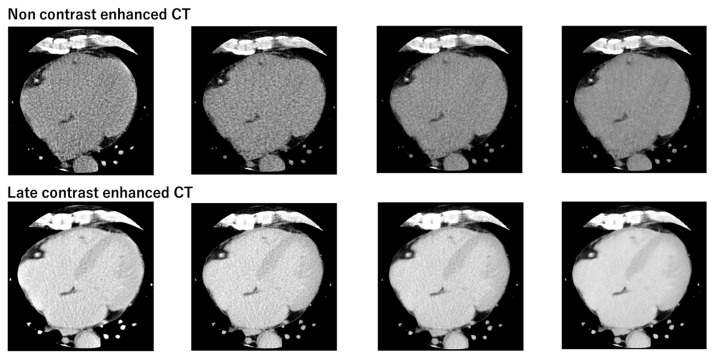
Comparison of non-contrast and late contrast-enhanced CT images using four reconstruction methods.

**Figure 4:**
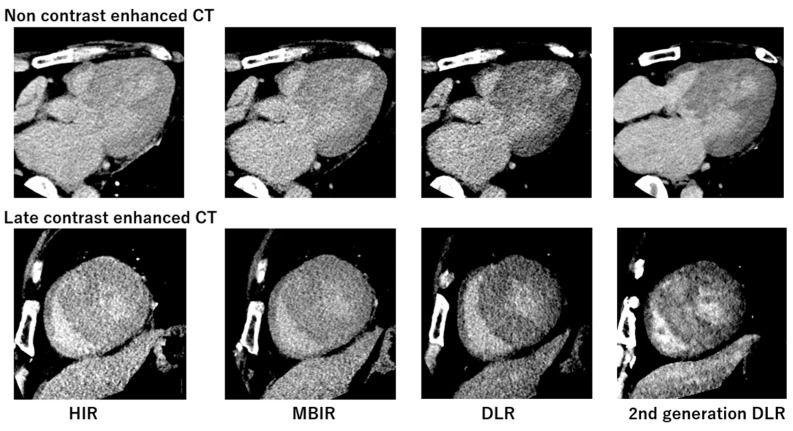
Comparison of non-contrast and late contrast-enhanced CT images using four reconstruction methods in a patient with hypertrophic cardiomyopathy.

**Figure 5:**
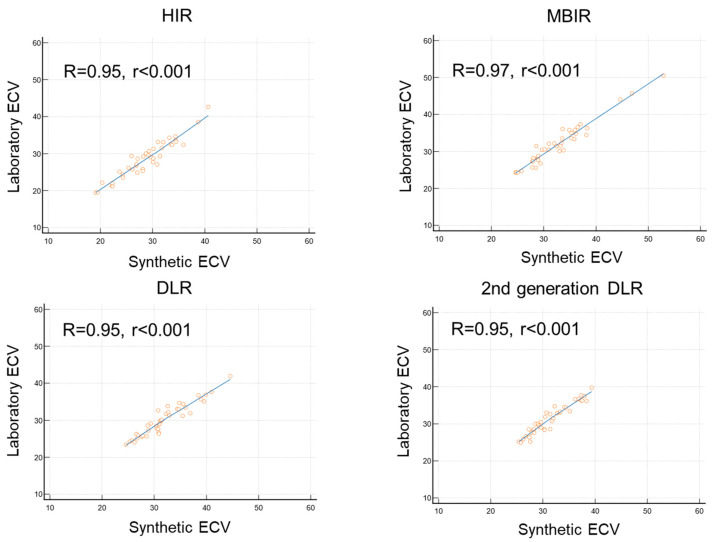
Correlation between synthetic ECV and laboratory ECV across four reconstruction methods in the validation cohort.

Figure 3 presents the non-contrast and late contrast-enhanced CT images for the four reconstruction methods, demonstrating that the second-generation DLR provides images with noticeably less noise. Figure 4 compares non-contrast and late contrast-enhanced CT images using four reconstruction methods in a patient with hypertrophic cardiomyopathy. The second-generation DLR clearly visualizes myocardial fibrosis in the septum. In the validation cohort, a high correlation was observed between synthetic ECV and laboratory ECV across all four reconstruction methods—HIR, MBIR, DLR, and second-generation DLR (r ≥ 0.95, *p* < 0.001, Figure 5). Among these methods, the bias and limit of agreement (LOA) in the Bland–Altman plot were the lowest with the second-generation DLR, indicating the highest accuracy.

**Figure 6:**
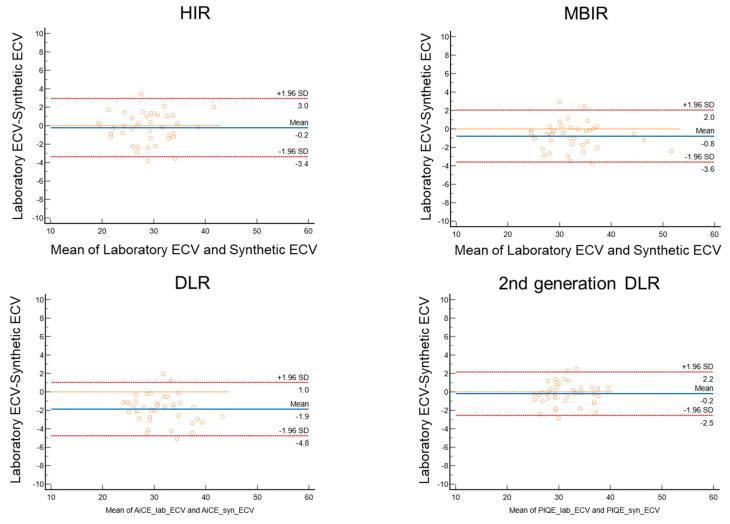
Bland–Altman plot between synthetic ECV and laboratory ECV across four reconstruction methods in the validation cohort.

Specifically, the bias and LOA were as follows: hybrid IR: bias = −0.21, LOA = 3.16; MBIR: bias = −0.79, LOA = 2.81; DLR: bias = −1.87, LOA = 2.90; and second-generation DLR: bias = −0.20, LOA = 2.35 (Figure 6). The ICC was 0.99 (95%CI: 0.98 to 0.99), indicating high reproducibility for calculating synthetic ECV by second-generation DLR.

## 4. Discussion

The main findings of this study are as follows: (1) Synthetic ECV, calculated using synthetic hematocrit without the need for blood tests, demonstrated a strong correlation with laboratory ECV across all four CT reconstruction methods. (2) Among these, the second-generation DLR achieved the most accurate quantification of synthetic ECV, with the lowest bias. These results underscore the potential utility of using second-generation DLR for evaluating synthetic ECV.

The evaluation of ECV is crucial for diagnosing heart disease and assessing risk stratification [12]. ECV can be measured non-invasively using cardiac CT [5,13,14]. However, this measurement process is complex, requiring venous blood sampling, image analysis, and offline ECV calculation, which poses significant challenges to its routine clinical application. Several previous studies have already demonstrated the efficacy of synthetic CT-ECV that does not require hematocrit values obtained from blood tests. Tribel et al. were the first, to our knowledge, to evaluate synthetic ECV without a blood sample, showing a strong correlation with laboratory ECV [7]. Kim et al. further assessed synthetic ECV using dual-energy CT, demonstrating a strong correlation not only with laboratory ECV but also with cardiac MRI ECV [8]. More recently, Mergen et al. evaluated synthetic ECV using virtual non-contrast CT from photon-counting CT [15]. In our study, we followed the method used by Tribel et al. [7] because we used a 320-row single-energy CT. We also focused on the technical aspect of CT, the reconstruction method. This is because the reconstruction technique is one of the most primitive techniques that contribute to the improvement of CT image quality. We compared four CT reconstruction methods and found that a recently developed DLR method, PIQE, provides the most accurate synthetic ECV.

The second-generation DLR refers to a newly developed and clinically introduced deep learning-based reconstruction algorithm. It has several advantages over previous reconstruction techniques. MBIR is not practical in clinical settings due to its long reconstruction time, which is caused by too many iterations. The first-generation DLR could only distinguish signal features from noise [10]. In contrast, the second-generation DLR improves cardiac image quality using deep learning technology, while achieving shorter reconstruction times. This advanced algorithm was developed using ultra-high-resolution CT data, which feature 160 slices with 0.25 mm collimation, as training data [16,17,18]. Ultra-high-resolution CT is characterized by its high noise reduction capability, and previous studies have reported that it improves the visibility of fine structures, such as coronary arteries, which is expected to enhance the diagnostic capability of coronary CTA [19]. Based on this background, we hypothesized that the second-generation DLR could also contribute to the assessment of left ventricular myocardial tissue characterization, leading us to design this study. As a result, we demonstrated that it offers high accuracy in the quantitative evaluation of synthetic ECV compared to other reconstruction techniques. Additionally, we believe that utilizing the second-generation DLR can help reduce radiation exposure, providing significant clinical benefits. With the capability to perform detailed ECV assessments via CT, there exists the potential to obviate the need for unnecessary myocardial biopsies and reduce reliance on time-intensive MRI evaluations. CT-ECV has demonstrated high efficacy in the detection of cardiac amyloidosis [5]. The use of synthetic ECV may enable the non-invasive and convenient identification of latent cardiac amyloidosis. This technology is particularly advantageous for patients with conditions commonly associated with comorbidities, such as aortic stenosis and atrial fibrillation, supporting the rationale for its use in conjunction with respective treatment plans, including transcatheter aortic valve implantation and ablation. Continued research in this domain is anticipated to yield further advancements.

## 5. Limitation

First, differences in tube voltage across different vendors and variations in reconstruction methods may impact the findings of this study. Some facilities may perform ECV measurements at 100 kVp or 80 kVp, and in such cases, the linear regression lines may not be consistent.

Second, avoiding misregistration in patients with arrhythmias or those who have difficulty holding their breath can be challenging. To mitigate this, it is crucial to provide thorough explanations to patients during the CT scan, understand the reconstruction phase, and make efforts to minimize the occurrence of misregistration. Furthermore, employing precise alignment techniques using workstations is also essential. Third, the clinical significance of synthetic ECV, particularly its relationship with the severity and risk of heart disease, was not investigated in this study. Additionally, while there have been many studies on synthetic ECV using MRI [20,21,22,23,24], the research using CT is still limited. To address these clinical questions, more large-scale studies are necessary.

Fourth, in this study, we have not examined the vendor variations for the calculation of synthetic ECV. Further studies were required to elucidate this point.

## 6. Conclusions

Synthetic ECV using second-generation DLR exhibited the least bias and narrowest LOA compared to laboratory ECV across the four reconstruction methods, indicating that second-generation DLR allows for more precise quantification.

## Figures and Tables

**Table 1:** Patients’ characteristics.

	Derivation Cohort (*n* = 40)	Validation Cohort (*n* = 40)
Male	60% (24/40)	63% (25/40)
Age, years	71 ± 12	67 ± 11
Interval between CT scan and blood test, days	3.0 ± 7.9	8.0 ± 6.7
Atrial fibrillation	2% (2/40)	0% (0/40)
eGFR, mL/min/1.73 m^2^	66.8 ± 15.2	61.20 ± 13.6

## Data Availability

Data are available upon reasonable request.

## References

[1] Mewton N., Liu C.Y., Croisille P., Bluemke D., Lima J.A. (2011). Assessment of myocardial fibrosis with cardiovascular magnetic resonance. J. Am. Coll. Cardiol..

[2] Haaf P., Garg P., Messroghli D.R., Broadbent D.A., Greenwood J.P., Plein S. (2016). Cardiac T1 Mapping and Extracellular Volume (ECV) in clinical practice: A comprehensive review. J. Cardiovasc. Magn. Reson..

[3] Flett A.S., Hayward M.P., Ashworth M.T., Hansen M.S., Taylor A.M., Elliott P.M., McGregor C., Moon J.C. (2010). Equilibrium contrast cardiovascular magnetic resonance for the measurement of diffuse myocardial fibrosis: Preliminary validation in humans. Circulation.

[4] Arbelo E., Protonotarios A., Gimeno J.R., Arbustini E., Barriales-Villa R., Basso C., Bezzina C.R., Biagini E., Blom N.A., de Boer R.A. (2023). 2023 ESC Guidelines for the management of cardiomyopathies. Eur. Heart J..

[5] Kato S., Misumi Y., Horita N., Yamamoto K., Utsunomiya D. (2024). Clinical Utility of Computed Tomography-Derived Myocardial Extracellular Volume Fraction: A Systematic Review and Meta-Analysis. JACC Cardiovasc. Imaging.

[6] Scully P.R., Patel K.P., Saberwal B., Klotz E., Augusto J.B., Thornton G.D., Hughes R.K., Manisty C., Lloyd G., Newton J.D. (2020). Identifying Cardiac Amyloid in Aortic Stenosis: ECV Quantification by CT in TAVR Patients. JACC Cardiovasc. Imaging.

[7] Treibel T.A., Fontana M., Steeden J.A., Nasis A., Yeung J., White S.K., Sivarajan S., Punwani S., Pugliese F., Taylor S.A. (2017). Automatic quantification of the myocardial extracellular volume by cardiac computed tomography: Synthetic ECV by CCT. J. Cardiovasc. Comput. Tomogr..

[8] Kim N.Y., Im D.J., Youn J.C., Hong Y.J., Choi B.W., Kang S.M., Lee H.J. (2022). Synthetic Extracellular Volume Fraction Derived Using Virtual Unenhanced Attenuation of Blood on Contrast-Enhanced Cardiac Dual-Energy CT in Nonischemic Cardiomyopathy. AJR Am. J. Roentgenol..

[9] Greffier J., Pastor M., Si-Mohamed S., Goutain-Majorel C., Peudon-Balas A., Bensalah M.Z., Frandon J., Beregi J.P., Dabli D. (2024). Comparison of two deep-learning image reconstruction algorithms on cardiac CT images: A phantom study. Diagn. Interv. Imaging.

[10] Kawai H., Motoyama S., Sarai M., Sato Y., Matsuyama T., Matsumoto R., Takahashi H., Katagata A., Kataoka Y., Ida Y. (2024). Coronary computed tomography angiographic detection of in-stent restenosis via deep learning reconstruction: A feasibility study. Eur. Radiol..

[11] Sawamura S., Kato S., Funama Y., Oda S., Mochizuki H., Inagaki S., Takeuchi Y., Morioka T., Izumi T., Ota Y. (2024). Evaluation of four computed tomography reconstruction algorithms using a coronary artery phantom. Quant. Imaging Med. Surg..

[12] Wong T.C., Piehler K., Meier C.G., Testa S.M., Klock A.M., Aneizi A.A., Shakesprere J., Kellman P., Shroff S.G., Schwartzman D.S. (2012). Association between extracellular matrix expansion quantified by cardiovascular magnetic resonance and short-term mortality. Circulation.

[13] Zhang H., Guo H., Liu G., Wu C., Ma Y., Li S., Zheng Y., Zhang J. (2023). CT for the evaluation of myocardial extracellular volume with MRI as reference: A systematic review and meta-analysis. Eur. Radiol..

[14] Muthalaly R.G., Tan S., Nelson A.J., Abrahams T., Han D., Tamarappoo B.K., Dey D., Nicholls S.J., Lin A., Nerlekar N. (2024). Variation of computed tomography-derived extracellular volume fraction and the impact of protocol parameters: A systematic review and meta-analysis. J. Cardiovasc. Comput. Tomogr..

[15] Mergen V., Ehrbar N., Moser L.J., Harmes J.C., Manka R., Alkadhi H., Eberhard M. (2024). Synthetic hematocrit from virtual non-contrast images for myocardial extracellular volume evaluation with photon-counting detector CT. Eur. Radiol..

[16] Takagi H., Tanaka R., Nagata K., Ninomiya R., Arakita K., Schuijf J.D., Yoshioka K. (2018). Diagnostic performance of coronary CT angiography with ultra-high-resolution CT: Comparison with invasive coronary angiography. Eur. J. Radiol..

[17] Motoyama S., Ito H., Sarai M., Nagahara Y., Miyajima K., Matsumoto R., Doi Y., Kataoka Y., Takahashi H., Ozaki Y. (2018). Ultra-High-Resolution Computed Tomography Angiography for Assessment of Coronary Artery Stenosis. Circ. J..

[18] Iwasawa T., Sato M., Yamaya T., Sato Y., Uchida Y., Kitamura H., Hagiwara E., Komatsu S., Utsunomiya D., Ogura T. (2020). Ultra-high-resolution computed tomography can demonstrate alveolar collapse in novel coronavirus (COVID-19) pneumonia. Jpn. J. Radiol..

[19] Orii M., Sone M., Osaki T., Ueyama Y., Chiba T., Sasaki T., Yoshioka K. (2023). Super-resolution deep learning reconstruction at coronary computed tomography angiography to evaluate the coronary arteries and in-stent lumen: An initial experience. BMC Med. Imaging.

[20] Censi S., Cimaglia P., Barbieri A., Naldi M., Ruggerini S., Brogneri S., Tonet E., Rapezzi C., Squeri A. (2021). Performance of Synthetic Extracellular Volume Fraction in Different Cardiac Phenotypes From a Prospective Cohort of Patients Referred for Cardiac Magnetic Resonance. J. Magn. Reson. Imaging.

[21] Kammerlander A.A., Duca F., Binder C., Aschauer S., Zotter-Tufaro C., Koschutnik M., Marzluf B.A., Bonderman D., Mascherbauer J. (2018). Extracellular volume quantification by cardiac magnetic resonance imaging without hematocrit sampling: Ready for prime time?. Wien. Klin. Wochenschr..

[22] Raucci F.J., Parra D.A., Christensen J.T., Hernandez L.E., Markham L.W., Xu M., Slaughter J.C., Soslow J.H. (2017). Synthetic hematocrit derived from the longitudinal relaxation of blood can lead to clinically significant errors in measurement of extracellular volume fraction in pediatric and young adult patients. J. Cardiovasc. Magn. Reson..

[23] Robison S., Karur G.R., Wald R.M., Thavendiranathan P., Crean A.M., Hanneman K. (2018). Noninvasive hematocrit assessment for cardiovascular magnetic resonance extracellular volume quantification using a point-of-care device and synthetic derivation. J. Cardiovasc. Magn. Reson..

[24] Thongsongsang R., Songsangjinda T., Tanapibunpon P., Krittayaphong R. (2021). Native T1 mapping and extracellular volume fraction for differentiation of myocardial diseases from normal CMR controls in routine clinical practice. BMC Cardiovasc. Disord..

